# Hepatokine Fetuin B expression is regulated by leptin-STAT3 signalling and associated with leptin in obesity

**DOI:** 10.1038/s41598-022-17000-w

**Published:** 2022-07-27

**Authors:** Dongmei Wang, Menghua Wu, Xiaofang Zhang, Long Li, Mingzhu Lin, Xiulin Shi, Yan Zhao, Caoxin Huang, Xuejun Li

**Affiliations:** 1 Department of Endocrinology and Diabetes, Xiamen Diabetes Institute, Fujian Key Laboratory of Translational Research for Diabetes, The First Affiliated Hospital of Xiamen University, School of Medicine, Xiamen University, Xiamen, 361003 China; 2 Department of Public Health and Medical Technology, Xiamen Medical College, Xiamen, 361023 China; 3 Institute of Drug Discovery Technology, Ningbo University, Ningbo, 315211 China

**Keywords:** Metabolic disorders, Molecular biology

## Abstract

Obesity is an expanding global public health problem and a leading cause of metabolic disorders. The hepatokine Fetuin B participates in regulating insulin resistance, glucose metabolism and liver steatosis. However, the mechanism underlying Fetuin B activation remains unclear. Our previous population-based study demonstrated a significant association between serum Fetuin B and body fat mass in an obese population, which indicates its potential in mediating obesity-related metabolic disorders. In the present study, we further revealed a significant correlation between Fetuin B and leptin, the classic adipokine released by expanding adipose tissue, in this obese population. Consistently, elevated Fetuin B and leptin levels were confirmed in diet-induced obese mice. Furthermore, an in vitro study demonstrated that the leptin signalling pathway directly activated the transcription and expression of Fetuin B in primary hepatocytes and AML12 cells in a STAT3-dependent manner. STAT3 binds to the response elements on *FetuB* promoter to directly activate *FetuB* transcription. Finally, the mediating effect of Fetuin B in insulin resistance induced by leptin was confirmed according to mediation analysis in this obese population. Therefore, our study identifies leptin-STAT3 as an upstream signalling pathway that activates Fetuin B and provides new insights into the pathogenic mechanisms of obesity-related metabolic disorders.

## Introduction

Obesity is an increasingly prevalent metabolic disease and an important global health threat that increases the risk of diabetes, cardiovascular disease, nonalcoholic fatty liver disease, hypertension and cancers^1^. Obesity develops as a result of excess caloric intake or inadequate energy expenditure. However, the mechanisms initiating obesity-related metabolic syndromes are complicated and have not been completely elucidated. As important metabolic organs, both adipose tissue and the liver play fundamental roles in regulating metabolic homeostasis. Moreover, as endocrine organs, they can also secrete adipokines and hepatokines to signal other organs to maintain metabolic homeostasis, which is termed “interorgan crosstalk”^2–7^. With the deepening of our understanding of the mechanisms of obesity, the involvement of interorgan crosstalk in regulating obesity-related metabolic disorders has received increasing attention.

Fetuin B protein is a secreted hepatokine belonging to the family of cysteine protease inhibitors; it shares 22% homology with Fetuin A, which is another well-known hepatokine^8–12^. Recently, the role of Fetuin B in obesity and its related metabolic disorders has been demonstrated. It has been revealed that Fetuin B levels are significantly elevated in diet-induced obese mice and patients with nonalcoholic fatty liver or diabetes^13,14^. In agreement with these findings, our previous study found that triglyceride levels and an insulin resistance risk index were positively correlated with serum Fetuin B levels in obese Chinese adults^15–17^. Moreover, obese mice that received recombinant Fetuin B showed exacerbated liver steatosis and glucose intolerance^13,18^. Consistently, in vitro studies have shown that exogenous Fetuin B impairs the insulin sensitivity of myocytes, hepatocytes and cardiomyocytes^13,19^. Therefore, Fetuin B is closely correlated with insulin resistance and potentially mediates obesity-related metabolic disorders. However, the precise mechanisms that trigger Fetuin B in obesity remain unclear.

The key characteristic of obesity is the expansion of white adipose tissue (WAT). Under overnutrition, adipose tissues store more lipids, adipocytes increase in size, and adipokines are secreted to signal other organs to achieve metabolic adaptation^5,6^. Leptin was the first discovered adipokine; it was discovered in 1994 by positional cloning. Leptin is predominantly produced by adipose tissue and was initially identified for its prominent action on the hypothalamus to control food intake, energy expenditure and body weight. Leptin works by binding to its receptor LepR, which has six different isoforms. Among these isoforms, the long isoform of leptin receptor, LepRb, is the only isoform that exerts signal transduction in vivo^20–22^. In addition to the central nervous system, leptin receptors are also broadly present in other peripheral tissues including skeletal muscle, liver, adipose and pancreas tissues^23–27^. Hepatic-specific deletion of the leptin receptor protected mice from age- and diet-related glucose intolerance by elevating plasma insulin and hepatic insulin sensitivity but did not alter glucose homeostasis in nonfasted mice on a normal diet^28^. Thus, an antagonizing effect of leptin on hepatic insulin signalling was suggested under hyperinsulinemia conditions. Taken together, as an adipokine, leptin is considered not only a marker of adipose expansion, but also the driving force of metabolic disorders by targeting diverse tissues in obesity^29,30^.

In our previous study, a cross-sectional study of 1318 obese adults who underwent serum Fetuin B and metabolism-related laboratory testing was conducted. Of note, serum Fetuin B was not significantly associated with body mass index (BMI), waist circumference or serum triglycerides, but it was, positively correlated with body fat mass (BFM)^15^. This raises the possibility that Fetuin B may be triggered by signals from expanding adipose tissue in obesity, such as, leptin. In the present study, we first explored the correlation between Fetuin B and leptin in serum from obese adults. We also conducted an in vivo study to confirm the increased circulating levels of Fetuin B and leptin in diet-induced obese mice compared with those fed normal chow. Moreover, an in vitro study was performed to demonstrate the effect of leptin activation on Fetuin B in hepatocytes and verify Fetuin B as a novel transcriptional target of STAT3. Finally, the mediation effects of serum Fetuin B on the association between serum leptin and HOMA-IR were confirmed in the above obese adults. Given this evidence, it is rational to propose that Fetuin B, a hepatokine involved in regulating insulin resistance and metabolism, is transcriptionally regulated by leptin released from adipose tissues. These findings provide new insights into the mechanisms of Fetuin B activation in hepatocytes and the pathogenic mechanisms of obesity-related metabolic disorders.

## Results

### Serum Fetuin B is positively associated with leptin in obesity

**Figure 1:**
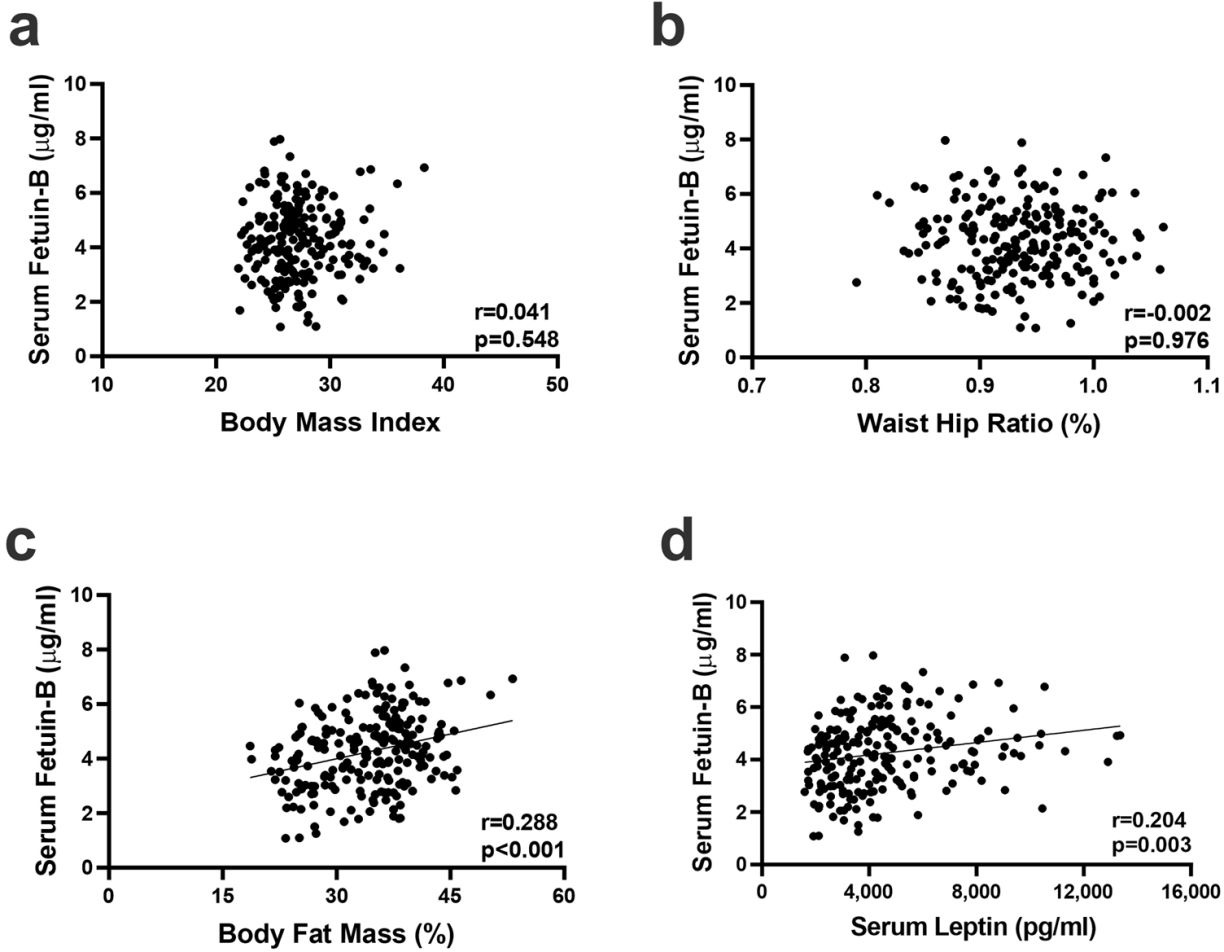
Correlations between serum Fetuin B and obesity indices and serum leptin in obese adults. The correlations between serum Fetuin B and the body mass index (BMI) (**a**), waist–hip ratio (WHR) (**b**), body fat mass (BFM) (**c**) and serum leptin (**d**) were assessed in 215 obese Chinese adults (n = 215). Pearson correlation coefficient was applied for correlation analysis. *P* values < 0.05 were considered statistically significant.

To explore the potential relationship between Fetuin B and obesity, 215 subjects were divided into three groups according to the serum Fetuin B tertiles, with concentrations (mean ± SD) as follows: Tertile 1, 2.752 ± 0.626 μg/mL; Tertile 2, 4.260 ± 0.358 μg/mL; Tertile 3, 5.721 ± 0.733 μg/mL. There were no significant differences in age, BMI, waist circumference, hip circumference, or waist–hip ratio (WHR) among the three groups. Of note, BFM increased significantly with serum Fetuin B concentrations (32.407 ± 6.454, 33.742 ± 6.551, and 36.269 ± 5.711% in Tertiles 1, 2, and 3, respectively; *p* = 0.001) (Table 1). We further analysed the correlation between Fetuin B and three key obesity indices, BMI, WHR and BFM (Fig. 1a–c). Only BFM, but not BMI or WHR, was positively correlated with serum Fetuin B. These findings highlight the possibility that Fetuin B may be regulated by adipokines. Thus, we further determined serum leptin levels in the 215 obese adults. Serum Fetuin B was positively associated with leptin in obesity (Fig. 1d).

**Table 1:** Demographic and clinical characteristics of subjects according to the tertiles of serum Fetuin B﻿.

	Tertile 1	Tertile 2	Tertile 3	Total	*P*
Fetuin B (μg/mL)	2.752 ± 0.626	4.260 ± 0.358	5.721 ± 0.733	4.252 ± 1.350	< 0.001
**Demographics**					
No. subjects	70 (32.56)	73 (33.95)	72 (33.49)	215 (100)	
Male (%)	33 (47.14)	25 (34.25)	13 (18.06)	71 (33.02)	
Female (%)	37 (52.86)	48 (65.75)	59 (81.94)	144 (66.98)	
Age (years)	54.028 ± 7.858	53.667 ± 6.871	52.681 ± 7.221	53.456 ± 7.313	0.523
**Clinical characteristics**					
BMI	27.341 ± 2.889	27.067 ± 2.960	27.402 ± 2.922	27.270 ± 2.914	0.766
Waist circumference (cm)	93.314 ± 6.537	93.028 ± 7.370	93.181 ± 7.637	93.173 ± 7.167	0.972
Hip circumference (cm)	99.979 ± 5.026	99.296 ± 5.924	100.092 ± 6.451	99.789 ± 5.813	0.679
Waist–hip ratio	0.934 ± 0.048	0.938 ± 0.053	0.932 ± 0.050	0.934 ± 0.050	0.792
Body fat (%)	32.407 ± 6.454	33.742 ± 6.551	36.269 ± 5.711	34.147 ± 6.423	0.001

Data are given as the mean ± SD or as n (%).

*BMI* body mass index.

**Figure 2:**
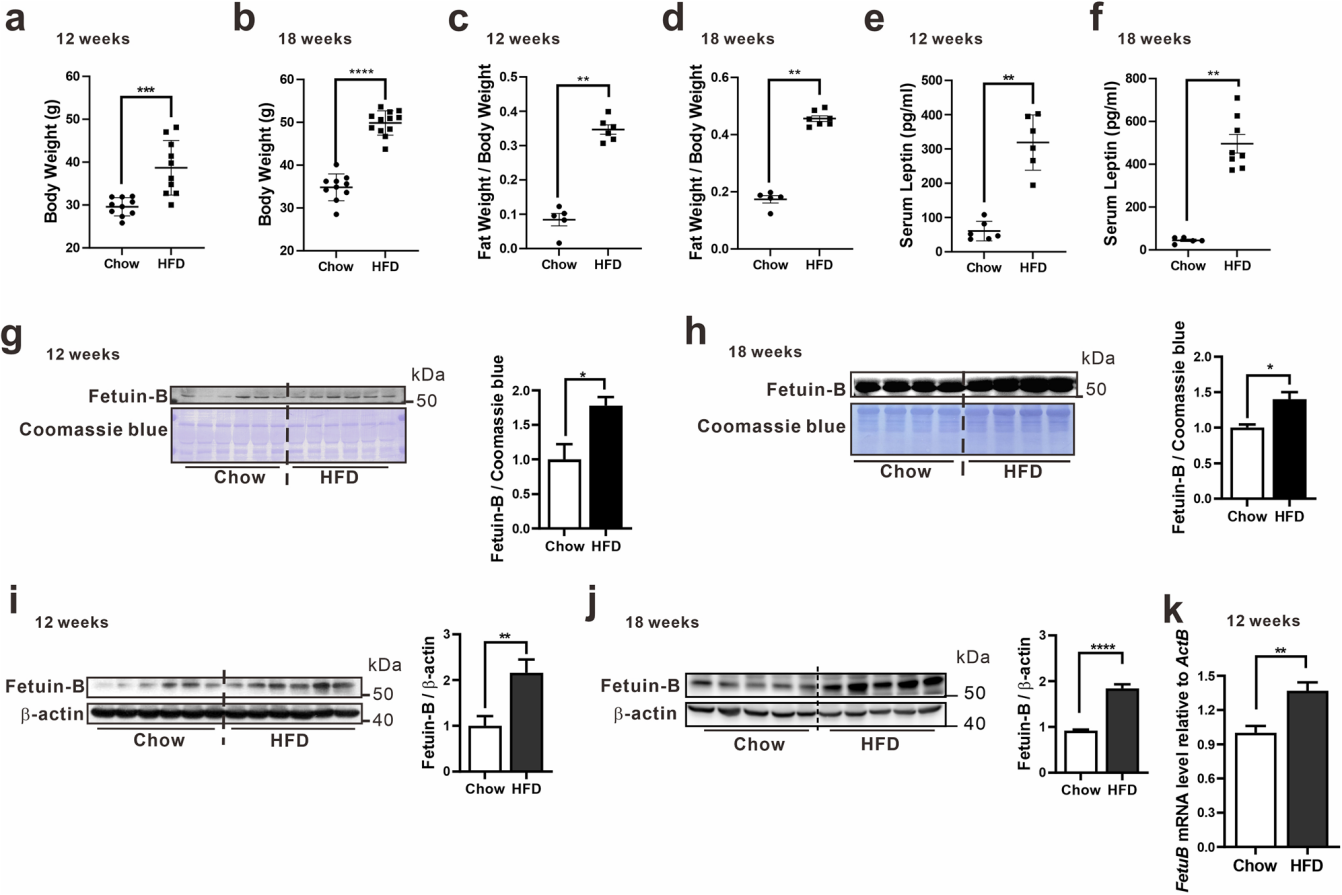
Expression of Fetuin B in HFD-induced obese mice. Body weight (**a**, **b**), body fat % (**c**, **d**), and serum leptin levels (**e**, **f**) were compared in mice fed chow with mice fed a HFD for 12 and 18 weeks (n = 5–12). The serum Fetuin B levels in mice fed chow or HFD for 12 and 18 weeks were analysed by western blot and normalized to Coomassie blue staining (**g**, **h**) (n = 4–6). The protein levels of Fetuin B in the liver of mice fed chow or HFD for 12 and 18 weeks were analysed by western blot and normalized to the loading control (β-actin) (**i**, **j**) (n = 5–6). The mRNA levels of Fetuin B in the livers of mice fed chow or a HFD for 12 weeks were analysed by qRT–PCR (k) (n = 5–6). Data are presented as mean ± SEM of all mice in each group. A nonparametric test (Mann–Whitney test) was applied. Significance is presented as *p < 0.05 compared with the chow group, **p < 0.01 compared with the chow group, ***p < 0.001 compared with the chow group, ****p < 0.0001 compared with the chow group. Original blots are presented in the Supplementary Information file.

To further confirm the association between Fetuin B and leptin in obesity, we established a mouse model of diet-induced obesity. C57BL/6 male mice were fed a high-fat diet (HFD) or normal chow and body weight increments were recorded (Supplemental Fig. 1a). Based on previous studies focusing on obesity and insulin resistance^31–35^, we first measured body fat, serum leptin and Fetuin B levels in mice fed a HFD or chow for 12 weeks. Mice fed a HFD for 12 weeks had significantly increased body weight, body fat (%), serum leptin levels, and Fetuin B levels in the serum and liver (Fig. 2a, c, e, g, i). Fasting blood glucose (FBG) levels, glucose tolerance and insulin sensitivity in mice fed a HFD or chow for 12 weeks were measured; insulin resistance was more severe in mice fed a HFD for 12 weeks (Supplemental Fig. 1b, d, e). To determine whether the upregulation of Fetuin B and leptin can persist during the progression of metabolic disorders by feeding the HFD for a longer period, we further measured body fat, FBG levels and serum leptin and Fetuin B levels in mice fed HFD or chow for 18 weeks. It was demonstrated that feeding the HFD for 18 weeks further increased body weight, body fat (%), FBG levels, serum leptin levels, and Fetuin B levels in the serum and liver (Fig. 2b, d, f, h, j, Supplemental Fig. 1c). In addition, lipid metabolism disorders in mice fed a HFD for 18 weeks were also determined (Supplemental Fig. 1f–i). Furthermore, we noticed that *FetuB* mRNA was significantly increased in mice fed a HFD for 12 weeks HFD mice compared to control mice, which suggests a transcriptional upregulation of Fetuin B in obesity (Fig. 2k). According to other publications, the upregulation of Fetuin B protein or mRNA levels was also demonstrated in obese mice fed a HFD for 6 weeks and 8 weeks^13,18^. It will be necessary to investigate the initiation of Fetuin B upregulation at earlier timepoints in the future. Taken together, these findings demonstrate that Fetuin B levels significantly increase with body fat and leptin levels and persist during the progression of obesity. Thus, these findings suggest the possibility that the expression of Fetuin B in the liver can be regulated by leptin.

### Leptin signalling pathway mediates upregulation of Fetuin B in hepatocytes

**Figure 3:**
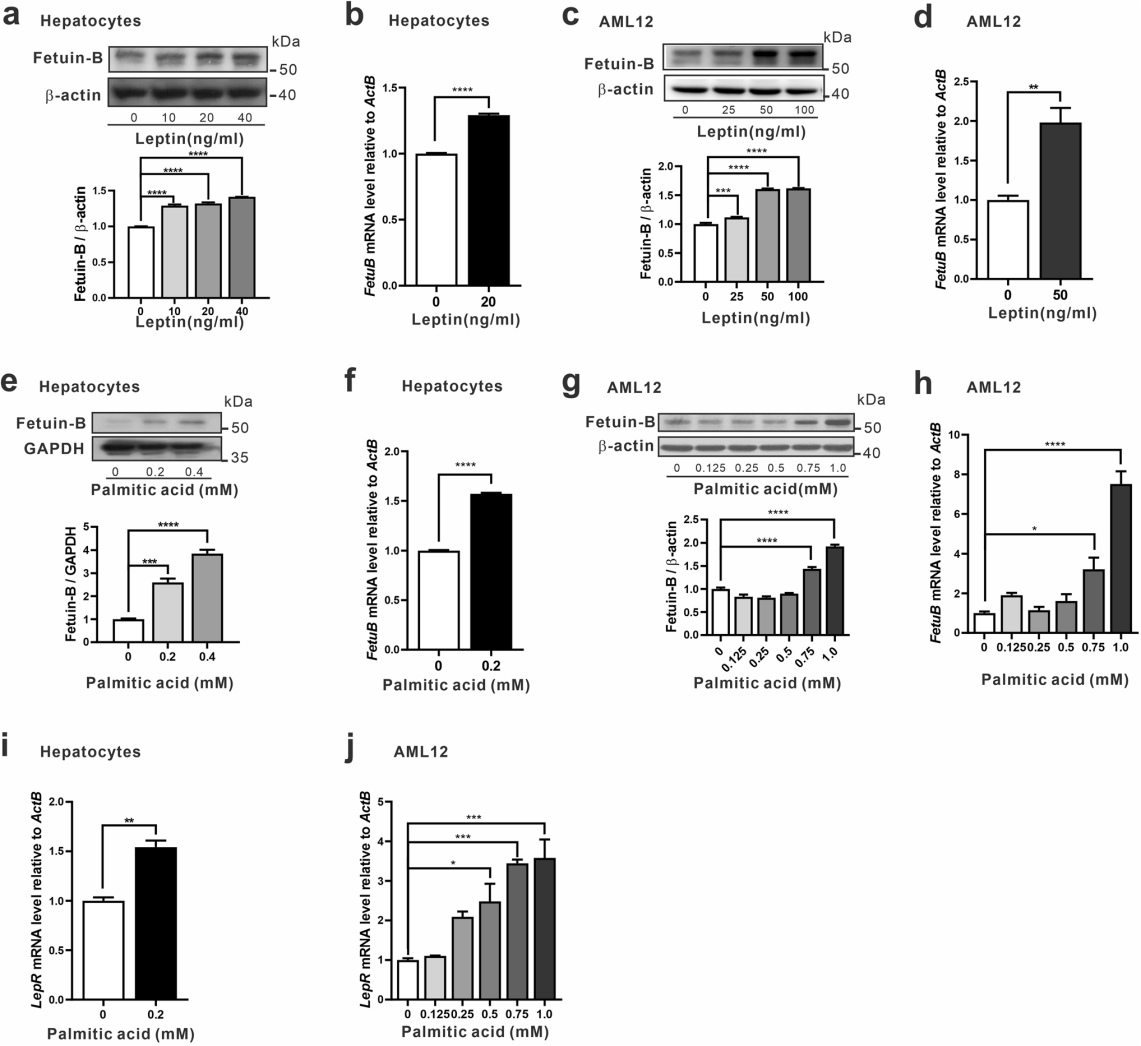
Effect of leptin and palmitic acid on Fetuin B expression in hepatocytes. The Fetuin B protein and mRNA levels were increased by treatment with leptin for 16 h in primary murine hepatocytes (**a**, **b**). The Fetuin B protein and mRNA levels were increased by treatment with leptin for 12 h in AML12 cells (**c**, **d**). Fetuin B protein and mRNA levels increased upon palmitic acid stimulation for 16 h in primary murine hepatocytes (**e**, **f**). The Fetuin B protein and mRNA levels were increased by palmitic acid stimulation for 12 h in AML12 cells. (**g**, **h**). The mRNA level of *LepRb* was analysed by qRT–PCR after palmitic acid stimulation in primary murine hepatocytes for 16 h (**i**) and AML12 cells for 12 h (**j**). qPCR data are presented as the mean ± SEM of two or three independent experiments. Western blot images are representative of two or more independent experiments. Quantitation of the representative western blot is presented as the mean ± SEM of triplicate measurements of the grey intensity ratio of Fetuin B/loading control. Original blots for independent replications for each experiment (n = 2–4) are presented in the Supplementary Information file. One-way analysis of variance (ANOVA) followed by Tukey’s multiple comparison test was used for experiments with three or more groups. *t* test was used to compare two groups. *p < 0.05 compared with the control group; **p < 0.01 compared with the control group; ***p < 0.001 compared with the control group, ****p < 0.0001 compared with the control group.

In light of the above in vivo study revealing the association between leptin and Fetuin B, we further conducted an in vitro study using primary murine hepatocytes and AML12 cells to investigate whether leptin could activate Fetuin B in hepatocytes. The doses of leptin in the study were chosen according to previous studies investigating effects of leptin on primary hepatocytes or AML12 in vitro, in which 10 ng/mL to 500 ng/mL were used more frequently^36–44^. It was demonstrated that leptin treatment increased Fetuin B protein levels in primary murine hepatocytes in a dose-dependent manner (Fig. 3a). In addition, Fetuin B mRNA levels were increased by leptin treatment (Fig. 3b). Consistently, both the protein and mRNA levels of Fetuin B in AML12 cells were increased by leptin treatment (Fig. 3c, d). These results demonstrate that exogenous leptin treatment could increase the expression and transcription of Fetuin B in hepatocytes, which suggests that Fetuin B is a transcriptional target of the leptin signalling pathway.

Increased levels of circulating or hepatic free fatty acids (FFAs) are another key hallmark of obesity. Previous work by Yang’s group showed that a mixture of oleate and palmitate could induce the mRNA and protein levels of Fetuin B in HepG2 cells^18^. Considering the increasing evidence showing more pronounced effects of inducing insulin resistance by palmitate compared to oleic acid, we treated both primary hepatocytes and AML12 cells with palmitic acid only^31,32^. The mRNA and protein levels of Fetuin B were significantly increased by palmitic acid treatment in both primary hepatocytes and AML12 cells (Fig. 3e–h). Of interest, palmitic acid treatment also increased the mRNA levels of the leptin receptor in hepatocytes in a dose-dependent manner (Fig. 3i, j). These findings further support the involvement of the leptin signalling pathway in mediating the activation of Fetuin B in hepatocytes.

### The leptin-induced upregulation of Fetuin B is STAT3 dependent

To elucidate the precise molecular mechanism by which leptin regulates Fetuin B, we evaluated the activation of STAT3, which is a classical downstream gene of leptin/LepRb signalling. The binding of leptin to LepRb results in the phosphorylation and the subsequent activation of JAK2. This activation leads to the recruitment and stimulation of various downstream pathways. Among these pathways, phosphorylated STAT3 proteins can dimerize and translocate to the nucleus to activate downstream genes such as suppressor of cytokine signalling 3 (SOCS3) and TIMP metallopeptidase inhibitor 1 (TIMP1)^45–48^. To verify the role of STAT3 in the regulation of Fetuin B by leptin, we measured STAT3 phosphorylation in AML12 cells upon leptin treatment.

**Figure 4:**
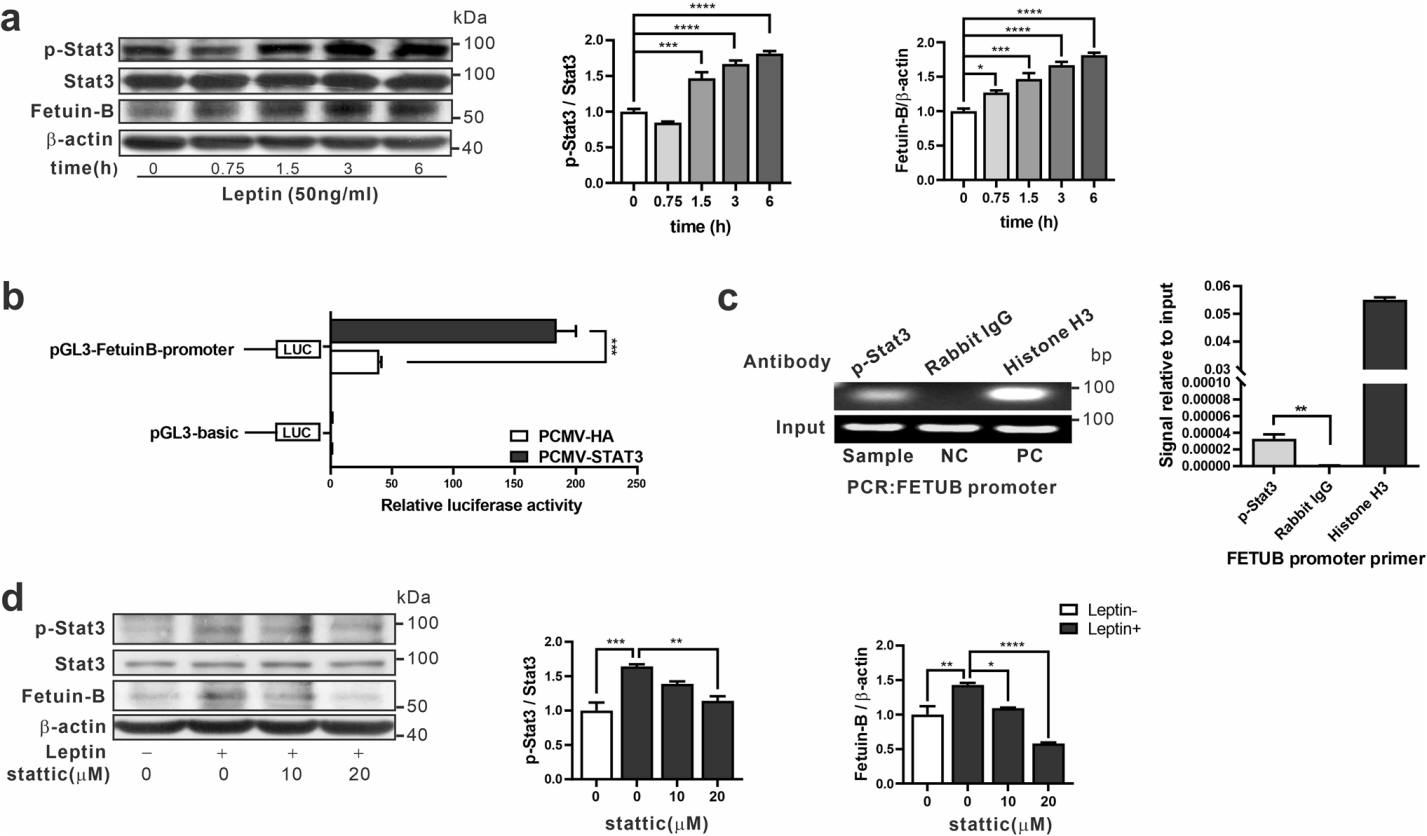
STAT3 phosphorylation mediates the transcription of *FetuB* in AML12 cells. STAT3 phosphorylation and Fetuin B protein expression levels in AML12 cells treated with leptin (50 ng/mL) for up to 6 h were analyzed by western blot (**a**). A Dual-Luciferase Reporter Assay was applied to determine *FetuB* promoter activity in AML12 cells by co-transfecting 100 ng luciferase reporter vector, 50 ng pRL-SV40-N and 100 ng pCMV-mSTAT3 or pCMV-HA. Firefly and Renilla luciferase activities were measured 36 h after transfection (**b**). ChIP assays were conducted to detect the binding of p-STAT3 to the *FetuB* promoter in AML12 cells treated with leptin treatment (50 ng/mL) for 12 h. PCR products were visualized by agarose gel electrophoresis and the efficiency of ChIP was calculated (**c**). STAT3 phosphorylation and Fetuin B levels in AML12 cells treated with or without leptin and Stattic treatments were analyzed by western blot (**d**). Data are presented as the mean ± SEM of three independent luciferase reporter assays and ChIP experiments. Quantitation of the representative western blot is presented as the mean ± SEM of triplicate measurements of grey intensities of targets. Original blots for independent replications for each experiment (n = 3) are presented in the Supplementary Information file. One-way analysis of variance (ANOVA) followed by Tukey’s multiple comparison test was used for experiments with three or more groups. *t* test was used to compare two groups. *p < 0.05 compared with the control group; **p < 0.01 compared with the control group; ***p < 0.001 compared with the control group, ****p < 0.0001 compared with the control group.

When AML12 cells were treated with 50 ng/mL leptin for 0.75 to 6 h, Fetuin B expression was significantly increased. Moreover, the phosphorylation level of STAT3 was significantly increased (Fig. 4a). Given these findings, we further determined whether STAT3 can directly activate Fetuin B at the transcriptional level. Bioinformatic prediction identified several putative STAT3-response elements in the region 2 kb upstream of the *FetuB* transcriptional start site. Thus, the mouse *FetuB* promoter (positions: –325 bp/ + 2000 bp) was cloned into the pGL3 basic backbone and transiently transfected into AML12 cells. Co-transfection with the STAT3 overexpression plasmid significantly enhanced the transcriptional activity of the luciferase reporter compared to that of the control group transfected with pCMV-HA (Fig. 4b). Furthermore, a ChIP assay was performed to determine whether phosphorylated STAT3 protein could be recruited to the *FetuB* promoter*.* As shown in Fig. 4c, the promoter fragment of *FetuB* can be amplified from the chromatin complex that is immunoprecipitated by the antibody against pStat3 in AML12 cells treated with leptin (50 ng/mL) for 12 h, but not with those using normal IgG. Thus, the binding of pStat3 to the *FetuB* promoter was confirmed.

Finally, we investigated whether the regulation of Fetuin B was STAT3-dependent using the STAT3-specific inhibitor Stattic. We pretreated AML12 cells with Stattic for 3 h in prior to leptin treatment for 12 h. The results demonstrated that Stattic inhibited STAT3 phosphorylation and the upregulation of Fetuin B by leptin dose-dependently (Fig. 4d). These results suggest that the STAT3 signalling pathway mediates the activation of Fetuin B by leptin.

### Leptin induces insulin resistance by increasing Fetuin B expression in obesity

**Figure 5:**
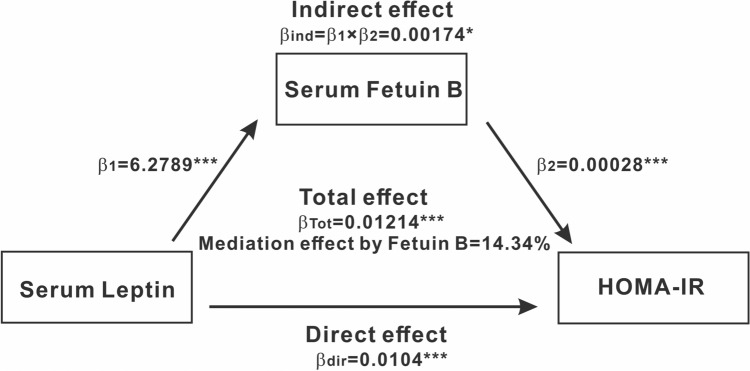
Leptin induces insulin resistance via Fetuin B. Mediation effects of serum Fetuin B on the association between serum leptin and HOMA-IR in obesity (n = 215). Sobel test was applied. Significance is presented as *p < 0.05, ***p < 0.001, and *p* < 0.05 for coefficients different from 0.

In conclusion, leptin directly induced the transcription and expression of Fetuin B in hepatocytes in a STAT3 phosphorylation-dependent manner. In combination with the antagonizing effect of leptin on hepatic insulin signalling under hyperinsulinemia conditions, such as obesity or ageing, determined by oral glucose tolerance tests and hyperinsulinemic-euglycemic clamp tests^28,30^, it is suspected that leptin/STAT3/Fetuin B may function as a novel adipose-liver axis in vivo to regulate obesity-induced insulin resistance that will ultimately lead to metabolic disorders. To verify the potential of Fetuin B in mediating leptin-induced insulin resistance in obesity, we performed mediation analysis in the 215 adults mentioned above. Figure 5 shows the mediation effect of serum Fetuin B on the association between serum leptin and the homeostasis assessment model for insulin resistance (HOMA-IR) in this population. The total effect of serum leptin on HOMA-IR as a standardized regression coefficient (βTot = 0.01214; *p* < 0.001) was estimated without serum Fetuin B in the model. β1 and β2 were used to calculate the indirect effect of serum Fetuin B (βind = 0.00174; *p* < 0.05). The percentage of the total effect mediated by serum Fetuin B was estimated to be at 14.34%. Therefore, these findings suggest that serum Fetuin B partially mediates the association between serum leptin and insulin resistance.

## Discussion

Obesity is an important risk factor for metabolic syndromes featuring insulin resistance and type 2 diabetes. However, the mechanisms linking obesity to metabolic disorders have not been completely elucidated. Fetuin B has been revealed as a hepatokine that is involved in regulating metabolic homeostasis and insulin resistance. The mechanisms that determine Fetuin B expression have not been well-defined. In the present study, we first demonstrated a significant correlation between BFM and serum Fetuin B levels in an obese population of 215 Chinese adults and proposed the hypothesis that Fetuin B can be regulated by signals from adipose tissue, such as adipokines. We further confirmed the correlation between leptin, the first identified adipokine, and Fetuin B in this population, and confirmed increased circulating levels of Fetuin B and leptin in diet-induced obese mice. Our in vitro study demonstrated that leptin could directly induce Fetuin B expression and transcription in a STAT3-dependent manner. Taken together, these results identify Fetuin B as a novel target of leptin in hepatocytes. These findings may provide new insight into adipose-liver crosstalk in the context of obesity and a more in-depth understanding of the effects of leptin on the liver that are mediated by Fetuin B.

Obesity refers to an excess of body fat, and it is closely associated with increased morbidity and mortality worldwide^49^. BMI, WHR and BFM are widely applied anthropometric parameters that are used to diagnose obesity and to assess the risk of morbidity and mortality. However, they show considerable differences. According to several population studies, BMI and WHR might be poor predictors for obesity and its metabolic risks in individuals^50,51^. Moreover, mounting evidence has indicated that body composition assessment could be a more accurate indicator of obesity and could more accurately evaluate metabolic risks^52–57^. In this study, we investigated a population of patients with central obesity, including males with waist circumferences greater than 90 cm and females with waist circumferences greater than 80 cm. We measured the BMI, WHR and BFM of 215 subjects to determine the relationship between these obesity indicators and Fetuin B. Of interest, only BFM, but not BMI or WHR, was positively correlated with Fetuin B. Importantly, as stated by our previous population-based study and other research groups, Fetuin B has been widely demonstrated to participate in regulating insulin resistance, glucose intolerance and steatosis in the liver^13,17–19,58^. Thus, the correlation between BFM and Fetuin B identified in the present study further supports the notion that BFM is a more convincing indicator for obesity-related metabolic risks.

As discussed above, the contribution of body fat to metabolic disorders has gained increasing attention^56,57^. It has become evident that adipokines contribute to interorgan crosstalk and regulate systemic metabolic homeostasis^2,5,6,59,60^. According to our study, leptin could activate Fetuin B and palmitate could activate Fetuin B and leptin receptor levels. These findings indicate the complexity of Fetuin B activation in vivo. In addition to what we have demonstrated in the present study, both FXR and Foxo1 have been revealed as the upstream transcription factors regulating Fetuin B expression in previous studies^19,61^. However, whether FXR or Foxo1 is involved in activating Fetuin B in the context of obesity requires further investigation. Moreover, an increasing number of studies have focused on the association between Fetuin B in females and metabolism-related disorders, gestational diabetes mellitus and polycystic ovary syndrome^62–64^. It would be interesting to further investigate how Fetuin B is regulated in female mice in the future and explore any potential involvement of sex-dependent regulation mechanisms.

The complexity of the actions of leptin is attributed to its multiple targeted tissues, sex and metabolic status^30,58,59^. As leptin is a major player in systemic metabolism, how it affects the liver locally has gained increasing interest. However, the regulatory mechanisms remain elusive. It has been demonstrated that leptin could play an important role in the progression of nonalcoholic steatohepatitis by promoting liver lipid decomposition and increasing insulin sensitivity in the early stage. It could also act as a pro-inflammatory and pro-fibrogenic factor in the advanced stage^27,65–68^. However, hepatic deficiency of the leptin receptor improved glucose tolerance and insulin sensitivity in aged and obese mice^28^. Moreover, the biological function of Fetuin B in promoting insulin resistance and glucose intolerance was consistent with the detrimental effects of leptin in the liver, as defined in aged or obese models. These findings further support the rationality of Fetuin B as a downstream target of leptin in the liver^13,15,17–19^. To verify the effect of Fetuin B on the association between leptin and insulin resistance, we performed mediation analysis in a population with central obesity. The results of mediation analysis showed that hyperleptinemia could induce insulin resistance, and Fetuin B partially mediated the increase in insulin resistance caused by leptin. Several studies have demonstrated that hepatocyte-specific deficiency of STAT3 can lead to insulin resistance and increased expression of gluconeogenic genes via the disruption of interleukin-6 (IL-6) signalling^69^. However, our data showed that STAT3 mediated the activation of Fetuin B, which was revealed to promote insulin resistance^13,15,17–19^. These findings emphasize the complexity of the STAT3 signalling network and suggest a possible competition between different triggering signals, such as leptin versus IL-6.

Taken together, our results demonstrated a correlation between Fetuin B and leptin in obese adults and identified Fetuin B as a transcriptional target of the leptin/STAT3 signalling pathway in hepatocytes. Furthermore, these results suggest a potential adipose-liver axis in regulating obesity-related metabolic disorders, which needs to be further confirmed in future studies. This study may provide new insights for the diagnosis, pathogenesis and therapeutic strategies of obesity-related metabolic diseases in the future.

## Materials and methods

### Human studies

Human studies were approved by the Human Research Ethics Committee of the First Affiliated Hospital of Xiamen University (Xiamen, China). All methods involving human participants were performed in accordance with the Declaration of Helsinki. Written informed consent was obtained from each participant. The present study was based on a baseline examination of 1523 adults who were over 40 years of age, as described in our previous publications^15,70^. Subjects, who had cancer, current treatment with systemic corticosteroids, biliary obstructive diseases, acute or chronic virus hepatitis, drug-induced liver diseases, total parenteral nutrition, autoimmune hepatitis, Wilson’s disease, or known hyperthyroidism or hypothyroidism, were excluded. Briefly, 215 adults were randomly chosen from the above population with complete information regarding serum Fetuin B levels and other examinations, and the leptin concentration was further determined. Body fat mass was quantified with the Hologic whole body DXA systems (Hologic Inc., Bedford, MA). Blood samples were obtained 12 h postfasting and analysed on a HITACHI 7450 analyser (HITACHI, Tokyo, Japan). All biochemical measurements were tested at the Xiamen Diabetes Institute.

### Animal studies

All animal experiments were approved by the Committee for Animal Research at Xiamen University (XMULAC20200139). All animal experiments were carried out in compliance with ARRIVE guidelines. All methods were carried out in accordance with relevant guidelines and regulations.

Male C57BL/6 mice were obtained from Shanghai SLAC Laboratory Animal Co. Ltd. (Shanghai, China). All animals were maintained in a controlled environment (temperature of 22 ± 2 °C, humidity at 58 ± 3%) and allowed access to diet and water ad libitum in 12 h light/dark cycles. Mice at 8-weeks of age were randomly grouped into the group fed normal chow (Chow) or the group fed high-fat diet (HFD, 60% fat calories, Research Diets, USA) for up to 20 weeks. Body fat mass was quantified with the EchoMRI-100H body composition analyser system (EchoMRI Inc., USA). When sacrificing mice, mice were deeply anaesthetized with pentobarbital sodium, and then blood was collected for further analysis. Afterwards, tissues were harvested and quickly frozen in liquid nitrogen for biochemical and molecular analysis. Animal experimental procedures were performed following the guidance approved by the Animal Ethics Committee of Xiamen University.

### Biochemical measures

Glucose tolerance tests were performed by intraperitoneal injection of D-glucose (Sigma–Aldrich, St. Louis, MO) at a dose of 1 mg/g body weight after a 16-h fast. For insulin tolerance tests, mice were injected with regular human insulin (Eli Lilly, Indianapolis, IN) at a dose of 1 unit/kg body weight after a 6-h fast. Blood glucose was determined using a portable blood glucose metre (LifeScan, Johnson & Johnson, New Brunswick, NJ). Serum TG levels were determined using a Triglyceride Quantification Colorimetric/Fluorometric Kit (K622, BioVision). Serum TC, LDL-c and HDL-c levels were determined by a multifunctional benchtop clinical chemistry analyser (BS-240vet, Mindray Animal Care, China).

### Cell isolation and culture

In the primary hepatocyte isolation experiment, anaesthetized mice were inserted with a catheter into the hepatic portal vein and perfused with Hank's Balanced Salt Solution (Invitrogen, CA, USA). After perfusion, the liver was perfused with collagenase buffer (0.375 μg/mL) (Sigma–Aldrich, Shanghai, China) five times. Then it was transferred to a dish, minced, filtered through a 75 μm mesh, and washed in DMEM three times to obtain hepatocytes. The hepatocytes were centrifuged in Percoll, resuspended in DMEM, stained with Trypan blue and counted. Isolated hepatocytes were plated into cell culture dishes and subsequent experiments were conducted 24 h later. AML12 cells were obtained from the Cell Bank of Chinese Academy of Sciences (Shanghai, China) and maintained in DMEM/F-12 (1:1) supplemented with fetal bovine serum (10%), ITS liquid media supplement (1%) and dexamethasone (40 ng/mL). The cells were cultured in culture plates at 37 °C and 5% CO_2_ in an incubator. In the experiments, hepatocytes were treated with palmitic acid (0.125, 0.25, 0.5, 0.75 or 1 mM), leptin (25, 50 or 100 ng/mL) or Stattic (5, 10 or 20 μM). Palmitic acid was purchased from Sigma–Aldrich (Shanghai, China). Recombinant murine leptin was purchased from Peprotech (Suzhou, China). Stattic was purchased from Selleck (Houston, USA).

### Quantitative reverse transcription polymerase chain reaction (qRT–PCR)

Total RNA was isolated from mouse liver tissues or cells using the RNAsimple Total RNA Kit (Tiangen, Beijing, China) according to the manufacturer’s instructions. Complementary DNA was synthesized from total RNA using a Fast Quant RT kit (Tiangen, Beijing, China) following the manufacturer’s instructions. Quantification of mRNA was carried out on a Roche LightCycler 480 Real–time PCR Machine using SYBR Green Master Mix (Roche, Shanghai, China). The relative gene expression was analysed by the ^ΔΔ^Ct (threshold cycle) method using *ActB* as a reference gene. The sequences of the primers used in this study were as follows:

**Table:** 

Quantification PCR	Primer sequences
*ActB* forward primer	5′-GGCTGTATTCCCCTCCATCG-3′
*ActB* reverse primer	5′-CCAGTTGGTAACAATGCCATGT-3′
*FetuB* forward primer	5′-GGCCCTGCTTACTATGTGGAA-3′
*FetuB* reverse primer	5′-GACCGTAGAACCTTGGCAAAT-3′
*LepR* forward primer	5′-TGGTCCCAGCAGCTATGGT-3′
*LepR* reverse primer	5′-ACCCAGAGAAGTTAGCACTGT-3′
**Luciferase assay**	
*Stat3* cloning forward primer	5′-ATGGCTCAGTGGAACCAGCTGCAGCAGC-3′
*Stat3* cloning reverse primer	5′-TTATTTCCAAACTGCATCAATGAAT-3′
*FetuB* promoter forward primer	5′-CGACGCGTTTCATAATCAATCTTTACTAACCAC-3′
*FetuB* promoter reverse primer	5′-CCCTCGAGCAGAAATCGCAGAAGGCT-3′
**ChIP assay**	
*FetuB* promoter forward primer	5′-GCCTTCTGCGATTTCTGGTG-3′
*FetuB* promoter reverse primer	5′-CTGTAAGCCACTCTGCCAAAT-3′

### Western blotting

Mouse hepatic tissues or cells were lysed in radioimmunoprecipitation (RIPA) buffer (Millipore, Beijing, China) with protease and phosphatase inhibitors (Roche, Shanghai, China). Mouse serum was diluted 1:100 with RIPA buffer containing protease and phosphatase inhibitors. The proteins were separated by SDS–PAGE and transferred onto PVDF membranes. Then the membranes were blocked in skim milk, cut according to a protein ladder (Thermo Fisher Scientific 26634 or 26616) at Mw 40 kDa, 50/55 kDa and 70 kDa to save the antibody and better normalize the expression of targets using the loading control from the same blot. Each cut membranes was incubated with antibodies against: Fetuin B (R&D Systems, #AF1275, 1:2000; Genetex, GTX112260, 1:2000), Tyr705 phosphorylated STAT3 (Abcam, #ab76315, 1:2000), total STAT3 (Abcam, #ab68153, 1:2000), GAPDH (Cell Signaling Technology, #5174, 1:1000) and β-actin (Sigma–Aldrich, #A2228, 1:5000) diluted with primary antibody dilution buffer (Beyotime Biotechnology, Shanghai, China). Normally membranes ranging from 70 to 140 kDa were incubated with pSTAT3 antibody and then stripped and blotted with STAT3 antibody. Membranes ranging from 55/50 to 70 kDa were incubated with Fetuin B antibody. Membranes ranging from 35/40 to 55/50 kDa were incubated with β-actin antibody. Membranes below 40 kDa were incubated with GAPDH antibody. Alternatively, membranes ranging from 35/40 to 70 kDa were incubated with Fetuin B antibody. Then they were stripped and incubated with β-actin antibody. Original blots and information of membrane cutting edges (by longer exposure or in bright field) for each experiment are presented in the Supplementary information file. Membranes were further incubated with secondary antibodies followed by washing steps and developed with ECL using X-ray films or equipment (ImageQuant LAS4000 mini, GE or ChemiScope 6200, Qinxiang, Shanghai). The relative expression levels of target proteins were quantitated using ImageJ software and normalized to the loading control on the same PVDF membrane or Coomassie blue staining.

### Coomassie blue staining

Coomassie blue staining was applied to determine loading amount of serum samples, which did not contain β-actin^71^. After immunodetection, the PVDF membranes were washed and then stained with Coomassie Blue Fast Staining Solution (Beyotime, Shanghai, China) for 15 min, washed with water and air-dried. The membrane was scanned in a flatbed scanner and the staining density for each complete lane was analysed.

### Enzyme-linked immunosorbent assay (ELISA)

The levels of human serum leptin (#ab179884), Fetuin B (#ab240684) and mouse serum leptin (#ab100718) were determined by ELISA (Abcam, Shanghai, China) according to the manufacturers’ instructions.

### Plasmid cloning and dual-luciferase reporter assay

A mouse *FetuB* promoter sequence was amplified from mouse genomic DNA by PCR with the following primers: forward 5′-CGACGCGTTTCATAATCAATCTTTACTAACCAC-3′ and reverse 5′-CCCTCGAGCAGAAATCGCAGAAGGCT-3′. The PCR product was further cloned into the pGL3 basic luciferase reporter vector (Beyotime, Shanghai, China) using MluI and XhoI to yield the reporter plasmid pGL3-FetuinB-promoter. The PCR product was amplified by forward 5′-ATGGCTCAGTGGAACCAGCTGCAGCAGC-3′ and reverse 5′-TTATTTCCAAACTGCATCAATGAAT-3′ and inserted into the pCMV-HA plasmid to construct the pCMV-mSTAT3 overexpression plasmid. The plasmid pRL-SV40-N was purchased from (Beyotime, Shanghai, China).

In the experiment, luciferase reporter vector pRL-SV40-N was co-transfected with pCMV-mSTAT3 or pCMV-HA into AML12 cells in Opti-MEM (Gibco, Shanghai, China) reduced serum medium using Lipofectamine 3000 (Invitrogen, Shanghai, China). After 36 h, firefly and Renilla luciferase were measured by the Dual-Glo^®^ Luciferase Assay System (Promega, Beijing, China). The ratio of firefly/Renilla luciferase was calculated for each sample and further normalized to the backbone transfection control.

### Chromatin immunoprecipitation assay

AML12 cells were treated with or without murine leptin (50 ng/mL) for 12 h and fixed in 1% formaldehyde (Cell Signaling Technology, Boston, USA). The chromatin immunoprecipitation (ChIP) assay was carried out according to the manufacturer’s protocol (Cell Signaling Technology, #9002). Rabbit immunoglobulin G (IgG) was used as a control for nonspecific immunoprecipitation of DNA and anti-Histone H3 (D2B12) XP® Rabbit mAb was used as a positive control. Anti-phospho-STAT3 (Tyr705) (Cell Signaling Technology, #9145) was purchased from Cell Signaling Technology. Immunoprecipitated protein–DNA crosslinking was reversed, and the DNA was purified for further qPCR analysis. ChIP enrichment efficiency was calculated for each sample (pStat3, IgG and H3 positive control) using the formula: percent input = 2% × 2 (CT 2% Input Sample − CT IP Sample). Alternatively, the enrichment efficiency of the pStat3 antibody was normalized to IgG (= 1). The qPCR products were separated with 1.5% agarose gel electrophoresis and imaged under ultraviolet light.

### Statistical analysis

Statistical calculations were performed with GraphPad Prism version 9.0 software for Windows. The results are expressed as the mean ± SEM. One-way analysis of variance (ANOVA) followed by Tukey's multiple comparison test was used for cell experiments with three or more groups. Nonparametric test (Mann–Whitney test) or *t* test was used for comparing two groups. Pearson correlation coefficient was applied for correlation analysis and Sobel test was used for mediation analysis in human studies. *P* values < 0.05 were considered statistically significant. The levels of significance indicated in the graphs are *p < 0.05; **p < 0.01; ***p < 0.001, ****p < 0.0001.

## Supplementary Information

Supplementary Figures.

## Data Availability

All data relevant to the study are included in the article.
