# Supplementary material for: Hepatokine Fetuin B expression is regulated by leptin-STAT3 signalling and associated with leptin in obesity
Source: Sci Rep. 2022 Jul 27;12:12869. doi: 10.1038/s41598-022-17000-w (PMC9329397; doi:10.1038/s41598-022-17000-w)
Supplement: Supplementary file 1 — Supplementary Figures. [file 41598_2022_17000_MOESM1_ESM.docx]

**Supplementary information**

**Title: Hepatokine Fetuin B expression is regulated by leptin-STAT3 signalling and associated with leptin in obesity**

Dongmei Wang^1,2 †^, Menghua Wu^1 †^, Xiaofang Zhang^1^, Long Li^1,3^, Mingzhu Lin^1^, Xiulin Shi^1^, Yan Zhao^1^, Caoxin Huang^1*^, Xuejun Li^1*^

**Supplemental Figure 1**


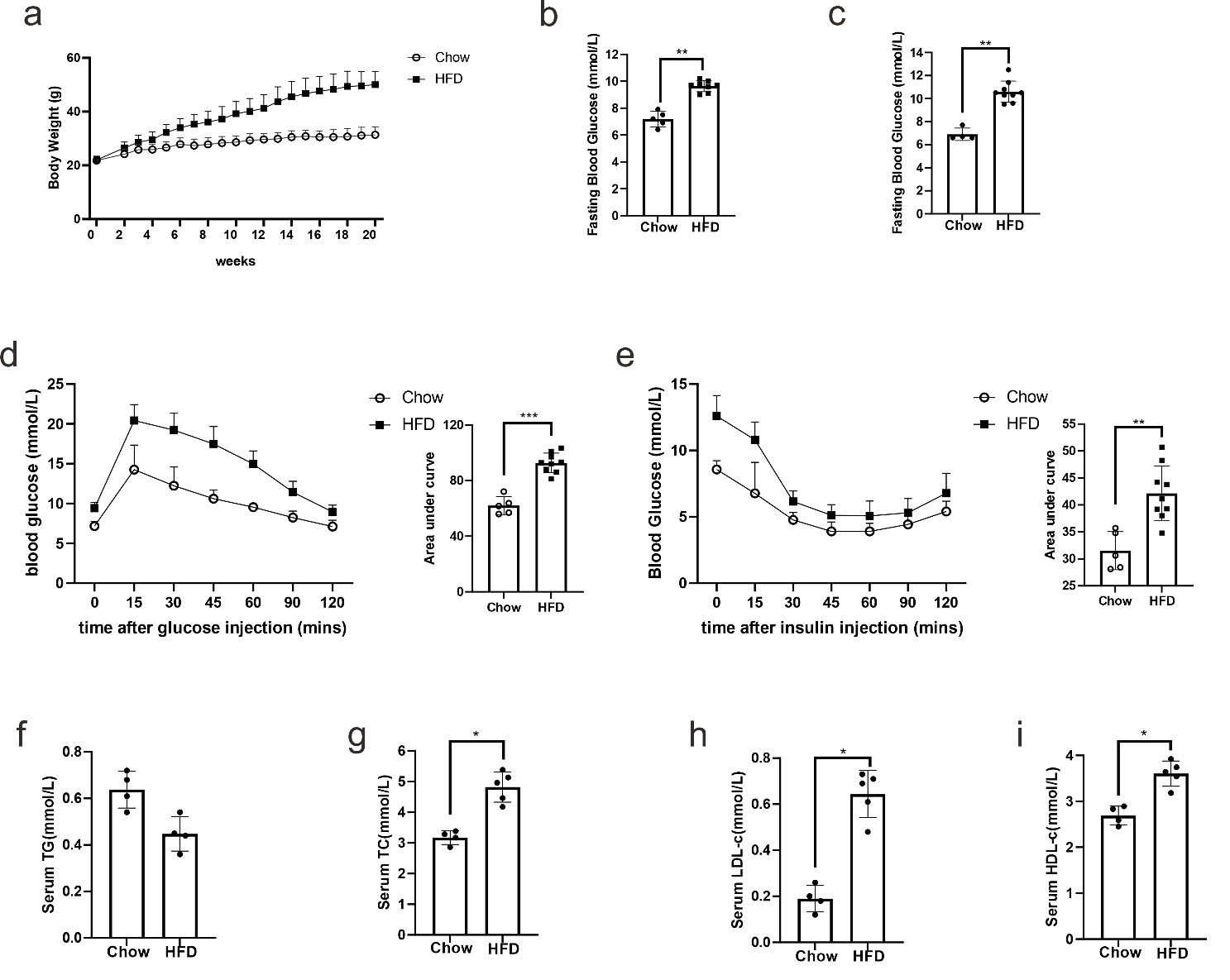


**Supplemental Figure 1. Biochemical comparisons of mice fed chow and a HFD.** The body weights of mice fed a HFD were significantly increased compared with those of mice fed chow (a). Fasting blood glucose levels were measured in mice fed chow or a HFD for 12 and 18 weeks (b-c) (n=5-9). The glucose tolerance test (d) and insulin tolerance test (e) were performed in mice fed chow or a HFD for 12 weeks (n=5-9). Serum TG, TC, LDL-c and HDL-c levels were determined in mice fed chow or a HFD for 18 weeks (f-i) (n=4-5). Data are presented as the mean ± SEM. Nonparametric test (Mann–Whitney test) was applied. Significance is presented as *p < 0.05 compared with the chow group, ** p < 0.01 compared with the chow group, and *** p < 0.001 compared with the chow group.

Raw data: Figure 2


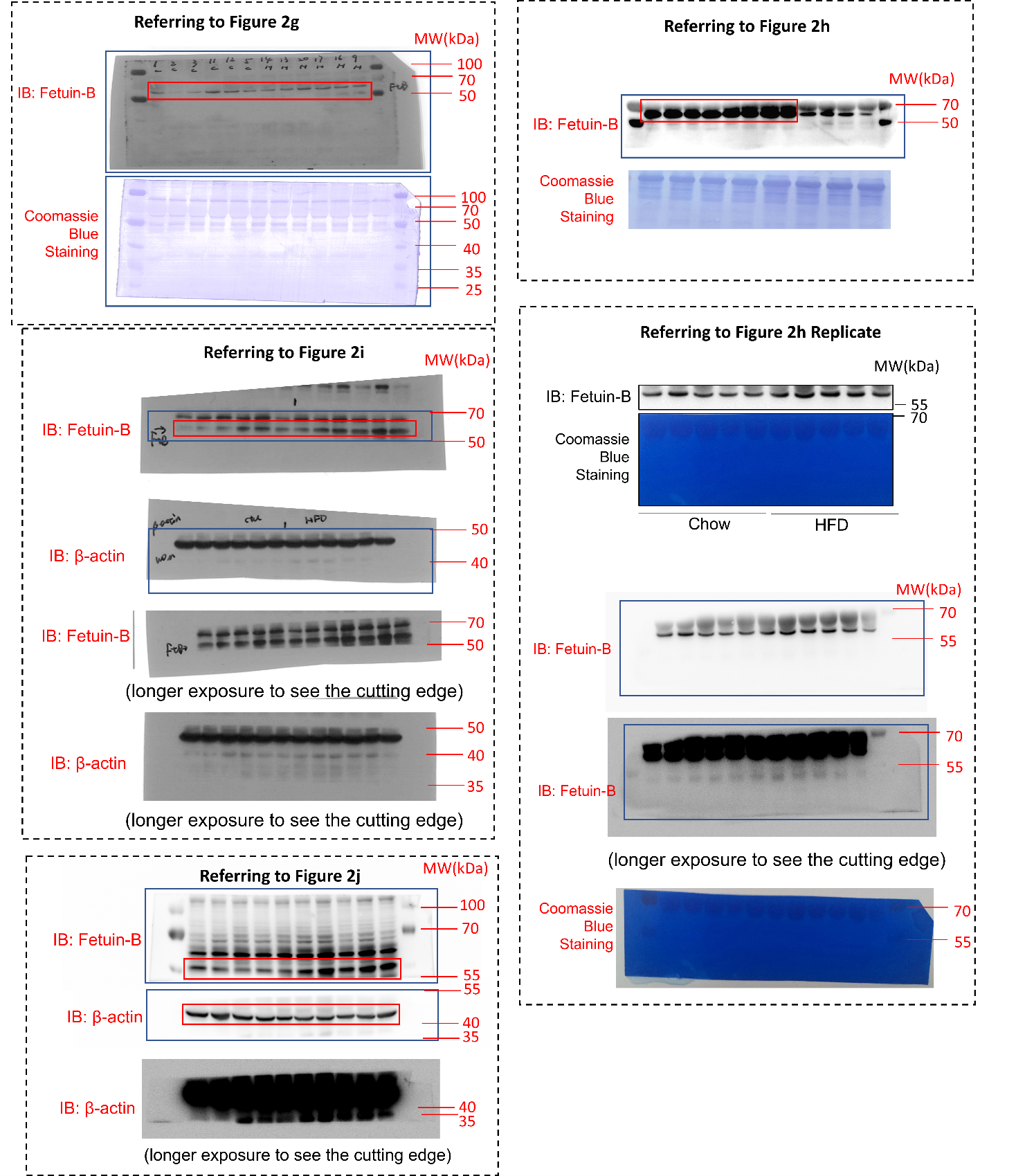


**Original blots of cropped western blot images and the related replicates (Figure 2 g-j).** To save the volume of antibody and to better normalize the expression of targets using the loading control from the same blot, the PVDF membrane was routinely cut according to the protein ladder (26616 or 26634, Thermo fisher) after the blocking stage. Membranes ranging from 55-70 kDa or 50-70 kDa were incubated with Fetuin B antibody. Membranes ranging from 35-55 or 35-50 kDa were incubated with β−actin antibody. A blue box was applied to outline the cutting edge of each membrane approximately. A red box was applied to outline the cropped regions for figures. Longer exposures available were presented to show the cutting edge of the membranes.

Raw data: Figure 3


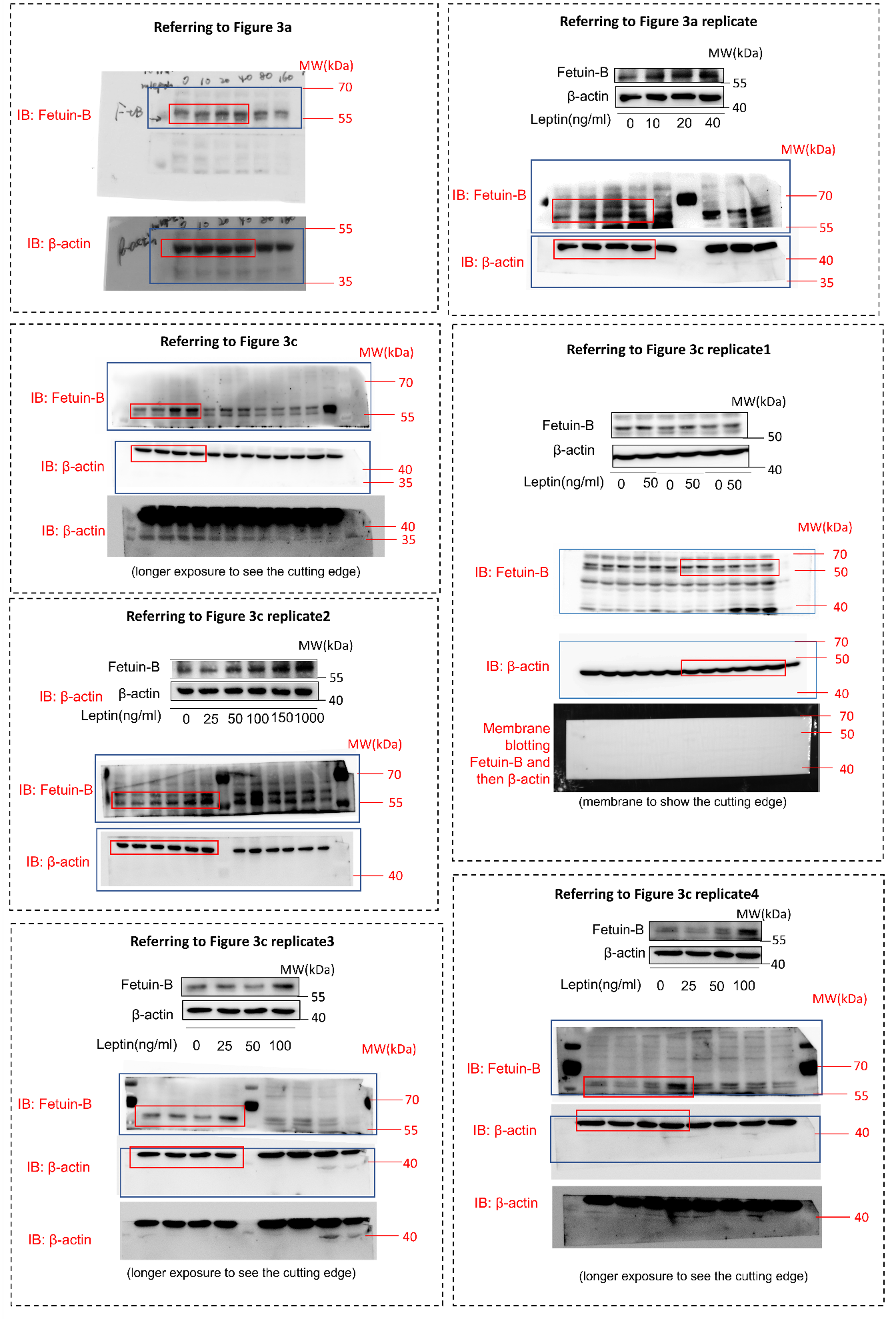


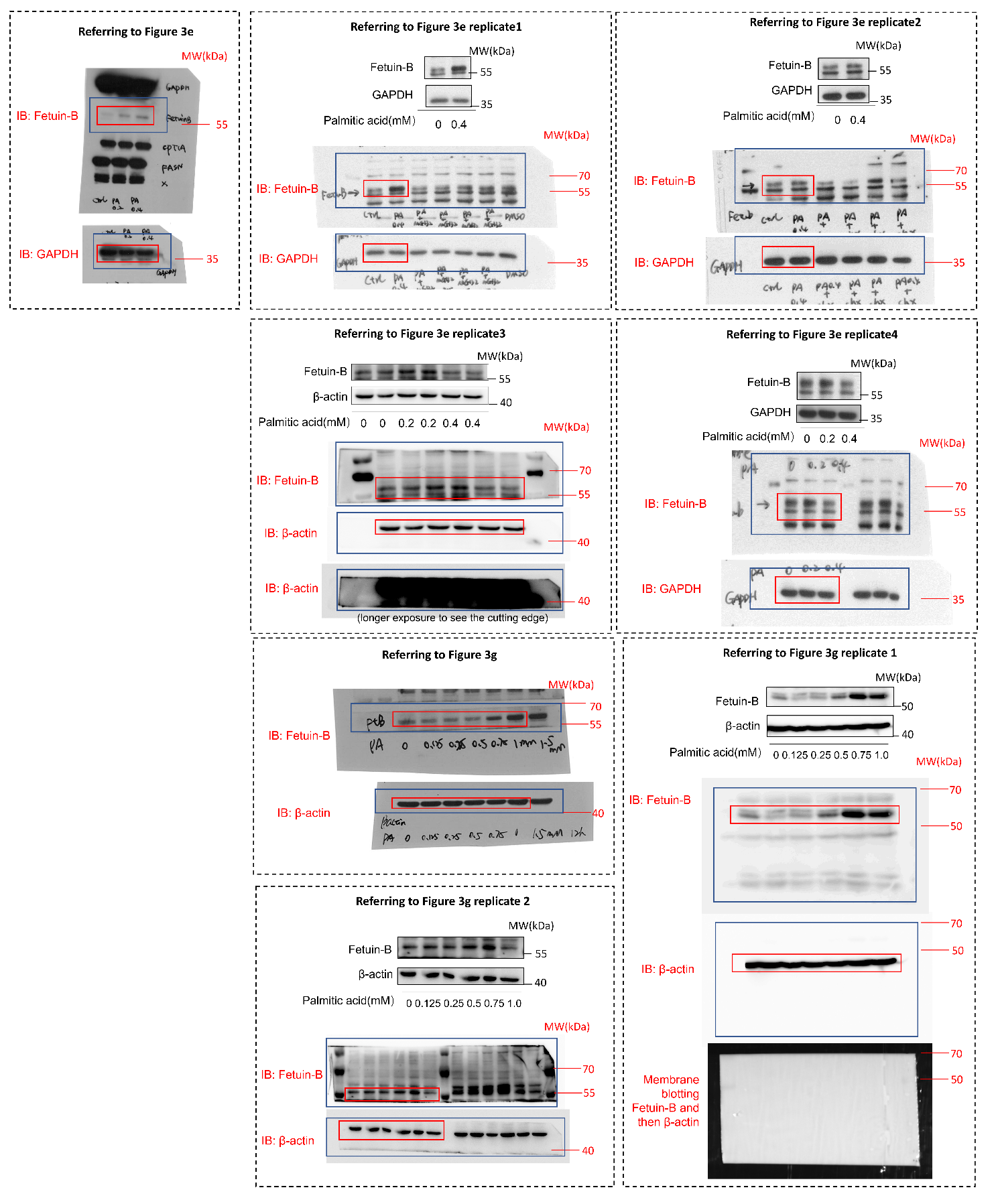


**Original blots of cropped western blot images and the related replicates (Figure 3 a, c, e, g).** To save the volume of antibody and to better normalize the expression of targets using the loading control from the same blot, the PVDF membrane was routinely cut according to the protein ladder (26616 or 26634, Thermo fisher) after blocking stage. For experiment using β−actin as loading control, membranes ranging from 55-70 kDa or 50-70 kDa were blotted with Fetuin B antibody and membranes ranging from 35-55 or 35-50 were blotted with β−actin antibody. For experiment using GAPDH as loading control, membranes ranging from 40-70 kDa were blotted with Fetuin B antibody and membranes below 40 kDa were blotted with GAPDH antibody. A blue box was applied to outline the cutting edge of each membrane approximately. A red box was applied to outline the cropped regions for figures. Longer exposures or membranes photographed at bright field were presented to show the cutting edge of the membranes.

Raw data: Figure 4

_
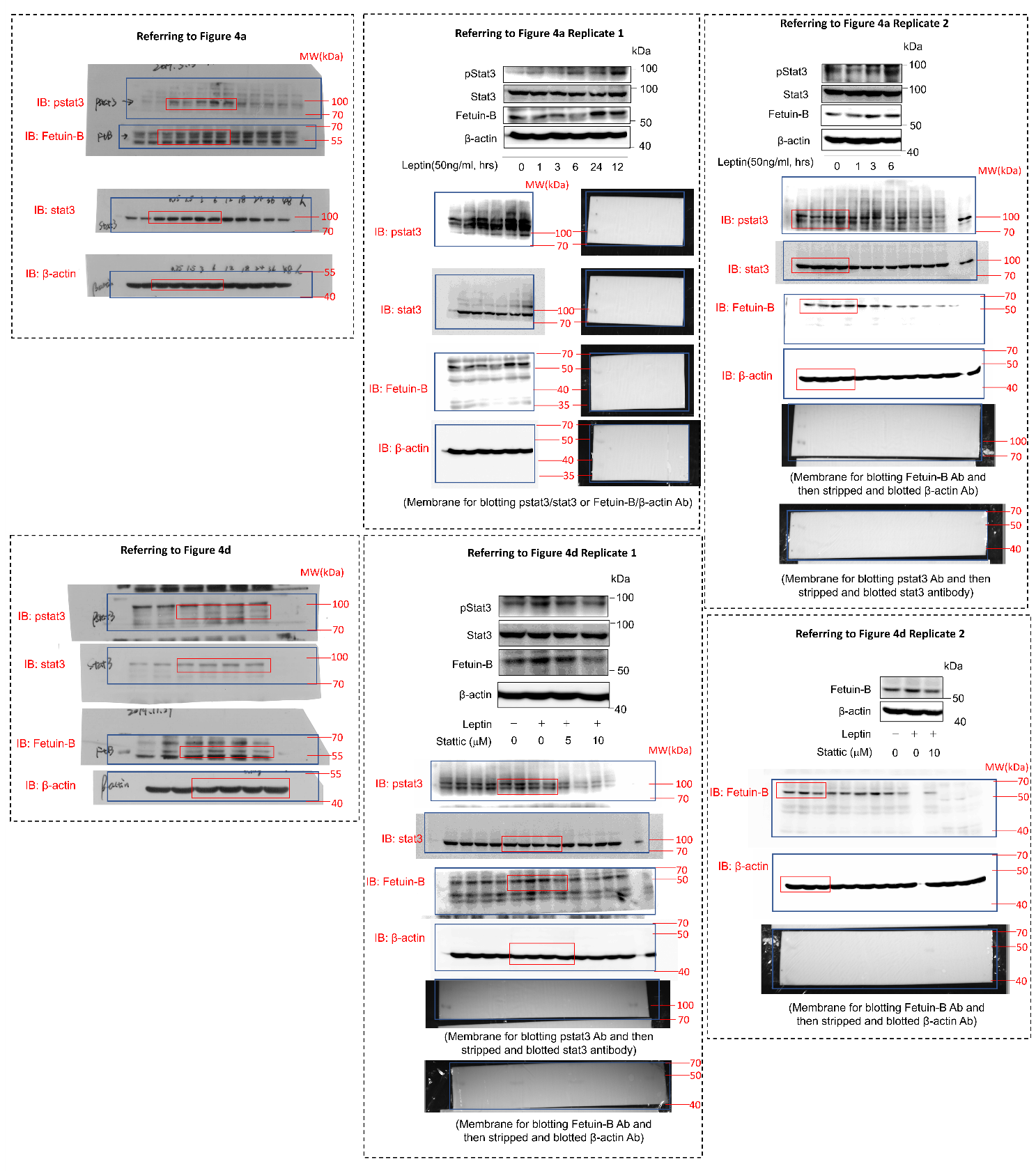
_

**Original blots of cropped western blot images and the related replicates (Figure 4 a, d).** To save the volume of antibody and to better normalize the expression of targets using the loading control from the same blot, the PVDF membrane was routinely cut according to the protein ladder (26616 or 26634, Thermo fisher) after blocking stage. Membranes ranging from 70-140 kDa were incubated with pStat3. Then these membranes were stripped and incubated with Stat3 antibody. Membranes ranging from 55-70 kDa or 50-70 kDa were blotted with Fetuin B antibody. Membranes ranging from 40-55/50 kDa were blotted with β−actin antibody. Alternatively, membranes ranging from 35/40-70kDa were first incubated with Fetuin B antibody and then stripped and incubated with β−actin antibody. A blue box was applied to outline the cutting edge of each membrane approximately. A red box was applied to outline the cropped regions for figures. Longer exposures or membranes photographed at bright field were presented to show the cutting the edge of membranes.
